# Circulating TGF-****β****1, Glycation, and Oxidation in Children with Diabetes Mellitus Type 1

**DOI:** 10.1155/2012/510902

**Published:** 2012-09-26

**Authors:** Vladimír Jakuš, Michal Sapák, Jana Kostolanská

**Affiliations:** ^1^Institute of Medical Chemistry, Biochemistry and Clinical Biochemistry, Faculty of Medicine, Comenius University, Sasinkova 2, Bratislava 81108, Slovakia; ^2^Department of Immunology, Faculty of Medicine, Comenius University, Sasinkova 2, Bratislava 81108, Slovakia; ^3^National Institute for Certified Educational Measurements, Žehrianska 9, Bratislava 85107, Slovakia

## Abstract

The present study investigates the relationship between diabetes metabolic control represented by levels of HbA1c, early glycation products-(fructosamine (FAM)), serum-advanced glycation end products (s-AGEs), lipoperoxidation products (LPO), advanced oxidation protein products (AOPP) and circulating TGF-**β** in young patients with DM1. The study group consisted of 79 patients with DM1 (8–18 years). 31 healthy children were used as control (1–16 years). Baseline characteristics of patients were compared by Student's *t*-test and nonparametric Mann-Whitney test (Statdirect), respectively. The correlations between the measured parameters were examined using Pearson correlation coefficient *r* and Spearman's rank test, respectively. A P value < 0.05 was considered as statistically significant. HbA1c was measured by LPLC, s-AGEs spectrofluorimetrically, LPO and AOPP spectrophotometrically and TGF-**β** by ELISA. Our results showed that parameters of glycation and oxidation are significantly higher in patients with DM1 than in healthy control. The level of serum TGF-**β** was significantly higher in diabetics in comparison with control: 7.1(3.6; 12.6) versus 1.6(0.8; 3.9) ng/mL. TGF-**β** significantly correlated with age and duration of DM1. There was not found any significant relation between TGF-**β** and parameres of glycation and oxidation. However, these results do not exclude the association between TGF-**β** and the onset of diabetic complications.

## 1. Introduction

Diabetes mellitus of Type 1 is one of the most frequent autoimmune diseases and is characterized by absolute or nothing short of absolute endogenous insulin deficiency which results in hyperglycemia that is considered to be a primary cause of diabetic complications. Diabetes mellitus leads to various chronic micro- and macrovascular complications. Diabetic nephropathy and cardiovascular disease are major causes of morbidity and mortality in patients with DM.

Persistent hyperglycemia is linked with glycation and glycoxidation. During glycation and glycoxidation, there are formed early, intermediate, and advanced glycation products (AGEs). Accumulation of AGEs has several toxic effects and takes part in the development of diabetic complications [1–3], such as nephropathy [4], neuropathy, retinopathy, and angiopathy [5]. Higher plasma levels of AGEs are associated also with incident cardiovascular disease and all-cause mortality in DM1 [6]. AGEs are believed to induce cellular oxidative stress through the interaction with specific cellular receptors [7].

It has been suggested that the chronic hyperglycaemia in diabetes enhances the production of reactive oxygen species (ROS) from glucose autoxidation, protein glycation, and glycoxidation, which leads to tissue damage [8–10]. Also, cumulative episodes of acute hyperglycaemia can be source of acute oxidative stress. A number of studies have summarized the relation between glycation and oxidation [11]. Uncontrolled production of ROS often leads to damage of cellular macromolecules (DNA, lipids, and proteins).

Some oxidation products or lipid peroxidation products may bind to proteins and amplify glycoxidation-generated lesions. Lipid peroxidation of polyunsaturated fatty acids, one of the radical reaction *in vivo*, can adequately reflect increased oxidative stress in diabetes.

Advanced oxidation protein products (AOPPs) are formed during oxidative stress by the action of chlorinated oxidants, mainly hypochlorous acid and chloramines. In diabetes, the formation of AOPP is induced by intensified glycoxidation processes, oxidant-antioxidant imbalance, and coexisting inflammation [12]. AOPPs are supposed to be structurally similar to AGEs and to exert similar biological activities as AGEs, that is, induction of proinflammatory cytokines in neutrophils, as well as in monocytes, and adhesive molecules [13]. Accumulation of AOPP has been found in patients with chronic kidney disease [14]. Further possible sources of oxidative stress are decreased antioxidant defenses, or alterations in enzymatic pathways. AGEs and their receptor (RAGE) axis stimulate oxidative stress and generation and subsequently evoke fibrogenic reactions in renal tubular cells, thereby playing arole in diabetic nephropathy [15]. Growth factor TGF-*β*1 is one of profibrotic cytokines and is an important mediator in the pathogenesis of diabetic nephropathy [16, 17]. TGF-*β*1 stimulates the production of extracellular matrix components such as collagen-IV, fibronectin, and proteoglycans (decorin, biglycan). TGF-*β*1 may cause glomerulosclerosis and it is one of the causal factor in myointimal hyperplasia after baloon injury of carotid artery. It mediates angiotensin-II modulator effect on smooth muscle cell growth. Beside profibrotic activity, TGF-*β*1 has immunoregulatory function on adaptive immunity too. AGEs induce connective tissue growth factor-mediated renal fibrosis through TGF-*β*1-independent Smad3 signalling [18, 19].

The present study investigates the relationship between diabetes metabolic control represented by actual levels of HbA1c, early glycation products—(fructosamine (FAM)), serum-advanced glycation end products (s-AGEs), lipid peroxidation products (LPOs), advanced oxidation protein products (AOPPs), and circulating TGF-*β* in patients with DM1.

## 2. Materials and Methods

### 2.1. Patients and Design

The studied group consisted of 79 children and adolescents (8–18 years) with T1DM regularly attending the 1st Department of Pediatrics, Children Diabetological Center of the Slovak Republic, University Hospital, Faculty of Medicine, Comenius University, Bratislava. They had T1DM with duration at least for 5 years. The urine samples in our patients were collected 3 times overnight, microalbuminuria was considered to be positive when UAER was between 20 and 200 microgram/min. No changes (fundus diabetic retinopathy) were found by the ophthalmologist examining the eyes in subject without retinopathy. Diabetic neuropathy was confirmed by EMG exploration using the conductivity assessment of sensor and motor fibres of peripheral nerves. The controls file consists of 31 healthy children (1–16 years). The samples of EDTA capillary blood were used to determine of HbA1c and serum samples were used to determine of FAM, s-AGEs, LPO, and AOPP. The samples of serum were stored in −18°C/−80°C.

### 2.2. Determination of UAER

UAER was determined by means of immunoturbidimetric assay (Cobas Integra 400 Plus, Roche, Switzerland), using the commercial kit 400/400 Plus. The assay was performed as a part of patients routine monitoring in Department of Laboratory Medicine, University Hospital, Bratislava.

### 2.3. Determination of Fructosamine

For the determination of fructosamine we used a kinetic, colorimetric assay and subsequently spectrophotometrical determination at wavelength 530 nm. We used 1-deoxy-1-morpholino-fructose (DMF) as the standard. Serum samples were stored at −79°C and were defrost only once. This test is based on the ability of ketoamines to reduce nitroblue tetrazolium (NBT) to a formazan dye under alkaline conditions. The rate of formazan formation, measured at 530 nm, is directly proportional to the fructosamine concentration. Measurements were carried out in one block up to 5 samples. To 3 mL of 0.5 mmol/L NBT were added 150 microliters of serum and the mixture was incubated at 37°C for 10 minutes. The absorbance was measured after 10 min and 15 min of incubation at Novaspec analyzer II, Biotech (Germany).

### 2.4. Determination of Glycated Hemoglobin HbA1c

HbA1c was determined from EDTA capillary blood immediately after obtained by the low pressureliquid chromatography (LPLC, DiaSTAT, USA) in conjunction with gradient elution. Before testing hemolysate is heated at 62–68°C to eliminate unstable fractions and after 5 minutes is introduced into the column. Hemoglobin species elute from the cation exchange column at different times, depending on their charge, with the application of buffers of increasing ionic strength. The concentration of hemoglobins is measured after elution from the column, which is then used to quantify HbA1c by calculating the area under each peak. Instrument calibration is always carried out when introducing a new column set procedure (Bio-RAD, Inc., 2003).

### 2.5. Determination of Serum AGEs

Serum AGEs were determined as AGE-linked specific fluorescence, serum was diluted 20-fold with deionized water, the fluorescence intensity was measured after excitation at 346 nm, at emission 418 nm using a spectrophotometer Perkin Elmer LS-3, USA. Chinine sulphate (1 microgram/mL) was used to calibrate the instrument. Fluorescence was expressed as the relative fluorescence intensity in arbitrary units (A.U.).

### 2.6. Determination of Serum Lipoperoxides

Serum lipid peroxides were determined by iodine liberation spectrophotometrically at 365 nm (Novaspec II, Pharmacia LKB, Biotech, SRN). The principle of this assay is based on the oxidative activity of lipid peroxides that will convert iodide to iodine. Iodine can then simply be measured by means of a photometer at 365 nm. Calibration curves were obtained using cumene hydroperoxide. A stoichiometric relationship was observed between the amount of organic peroxides assayed and the concentration of iodine produced [20].

### 2.7. Determination of Serum AOPP

AOPPs were determined in the plasma using the method previously devised by Witko-Sarsat et al. [21] and modified by Kalousová et al. [22]. Briefly, AOPPs were measured by spectrophotometry on a reader (FP-901, Chemistry Analyser, Labsystems, Finland) and were calibrated with chloramine-T solutions that in the presence of potassium iodide absorb at 340 nm. In standard wells, 10 microliters of 1.16 M potassium iodide was added to 200 microliters of chloramine-T solution (0–100 micromol/L) followed by 20 microliters of acetic acid. In test wells, 200 microliters of plasma diluted 1 : 5 in PBS were placed to cell of 9 channels, and 20 microliters of acetic acid was added. The absorbance of the reaction mixture is immediately read at 340 nm on the reader against a blank containing 200 microliters of PBS, 10 microliters of potassium iodide, and 20 microliters of acetic acid. The chloramine-T absorbance at 340 nm being linear within the range of 0 to 100 micromol/L, AOPP concentrations was expressed as micromoles per liter of chloramine-T equivalents.

### 2.8. Determination of Circulating TGF-*β*

Quantitative detection of TGF-*β* in serum was done by enzyme linked immunosorbent assay, using human TGF-*β*1 ELISA-kit (BMS249/2, Bender MedSystem). Brief description of the method: into washed, with anti-TGF-*β*1 precoated microplate were added prediluted (1 : 10) sera (100 microliters) and “HRP-Conjugate” (50 microliters) as a antihuman-TGF-*β*1 monoclonal antibody and incubated for 4 hour on a rotator (100 rpm). After microplate washing (3 times), “TMB Substrate Solution” (100 microliters) was added and was incubated for 10 minutes. Enzyme reaction was stopped by adding “Stop Solution” (100 microliters). The absorbance of each microwell was readed by HumaReader spectrophotometer (Human) using 450 nm wavelength. The TGF-*β*1 concentration was determined from standard curve prepared from seven TGF-*β*1 standard dilutions. Each sample and TGF-*β*1 standard dilution were done in duplicate.

### 2.9. Statistical Analysis

Shapiro-Wilk test was performed to the test the distribution of all continuous variables. Pearson's test with correlation coefficient *r* or Spearman's one with Spearman's rank correlation coefficient *R* in case of small count of variables was then used to association between parameters described within the text, in all studied patients. *P* values less than 0.05 were accepted as being statistically significant. All statistical analyses were carried out using Excel 2003, Origin 8 and BioSTAT 2009.

## 3. Results

### 3.1. Comparison of Clinical and Biochemical Parameters

Clinical and biochemical characteristics of the patients with DM1 without and with diabetic complications and controls are reported in Table 1.

**Figure 1:**
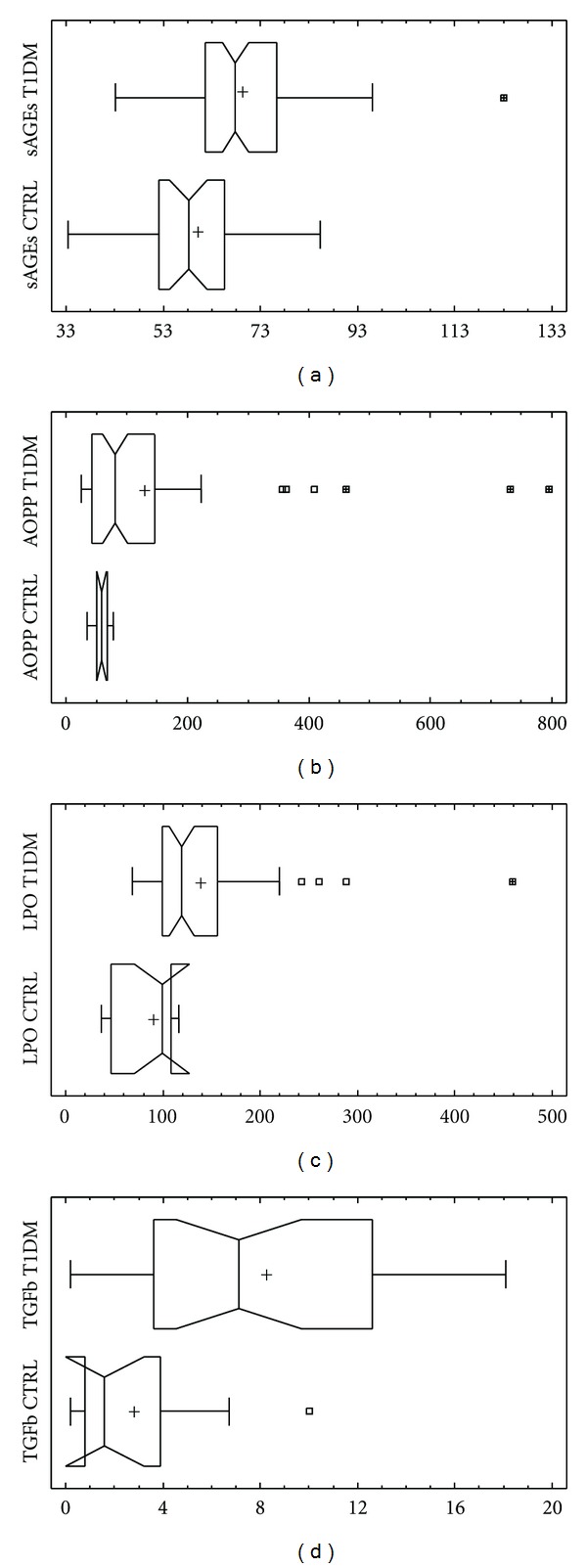
Comparison of (a) AGEs, (b) AOPP, (c) LPO and (d) TGF-**β**1 levels in diabetic patients and controls. Levels of AGEs are significantly higher in patients with DM1 than in healthy control (AGEs in serum 67.9 (61.6; 76.4) versus 58.2 (52.0; 65.0) A.U., *P* < 0.001*) (Figure 1(a)). Parameters of oxidative stress AOPP an LPO are significantly higher in patients with DM1 than in control (AOPP: 80.5 (44.9; 139.9) versus 51.5; 66.9) *μ*mol/L, *P* < 0.01*) (Figure 1(b)) (LPO: 119.0 (100.3; 156.3) versus 99.0 (67.0; 106.0) nmol/mL, *P* < 0.01*) (Figure 1(c)). The level of serum TGF-**β** was significantly higher in diabetics in comparison with control (7.1 (3.6; 12.6) versus 1.6 (0.8; 3.9) ng/mL, *P* < 0.01*) (Figure 1(d)). *-Mann Whitney test.

As shown in Table 1, there were significantly higher levels of AGEs (Figure 1(a)), AOPP (Figure 1(b)), LPO (Figure 1(c)) and TGF-beta (Figure 1(d)) in patients with DM1 than in healthy control.

### 3.2. Correlations between Measured Parameters

**Figure 2:**
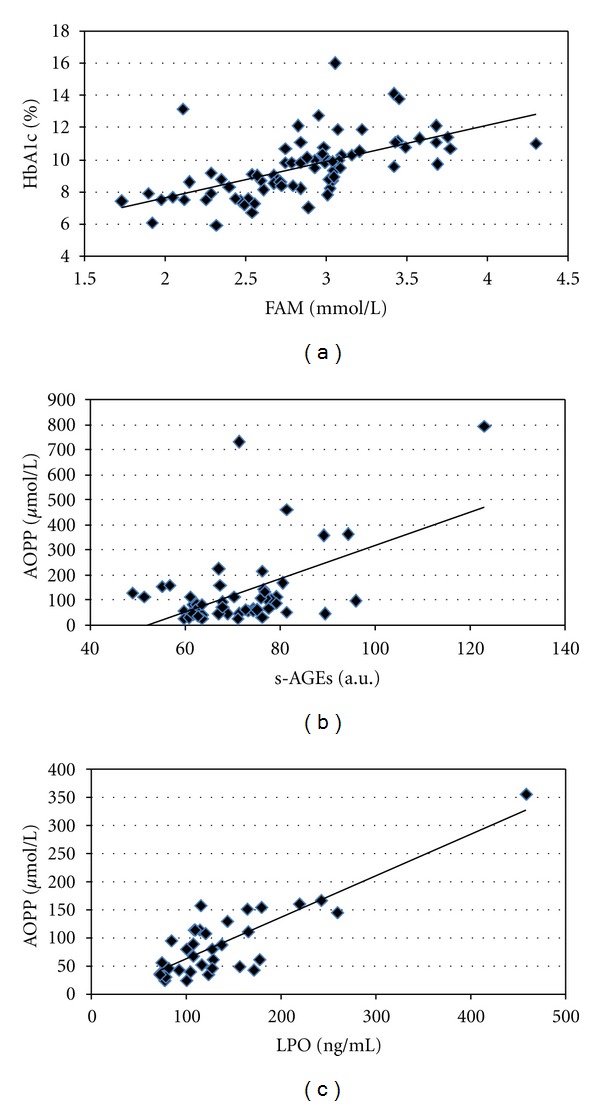
Significant correlations of (a) HbA1c and FAM (*r* = 0.601, *P* ≪ 0.001; *n* = 79), (b) AOPP and s-AGEs (*r* = 0.540, *P* ≪ 0.001; *n* = 54), and (c) AOPP and LPO (*r* = 0.833, *P* ≪ 0.001; *n* = 43) in all diabetic patients.

HbA1c significantly correlated with duration of DM1 (*r* = 0.294; *P* = 0.01) and with FAM (*r* = 0.601; *P* ≪ 0.001) (Figure 2(a)). S-AGEs significantly correlated with FAM (*r* = 0.368; *P* < 0.01).

AOPP has also significant correlation with FAM (*r* = 0.440; *P* ≪ 0.001), HbA1c (*r* = 0.455; *P* ≪ 0.001), and s-AGEs (*r* = 0.540; *P* ≪ 0.001) (Figure 2(b)).

LPO significantly correlated with FAM (*r* = 0.386; *P* < 0.01), with s-AGEs (*r* = 0.354; *P* = 0.02) and very strong with AOPP (*r* = 0.833; *P* ≪ 0.001) (Figure 2(c)).

TGF-**β** significantly correlated with age (*r* = 0.460; *P* = 0.01) and duration of DM1 (*r* = 0.379; *P* < 0.05). Relations with other parameters were not statistically significant.

## 4. Discussion

Many studies deal with the impact of glycative stress on the development of diabetic complications. We studied also glycative and oxidative stress parameters in regard to diabetic complications presence-absence and in with respect to glycemic compensation [23, 24]. In this work, we have focused on the study of relationship between clinical parameters, circulating markers of glycation, or oxidation and circulating cytokine TGF-*β* in young patients (children and adolescents) with DM1 without albuminuria (Table 1). Microalbuminuria is first clinical manifestation of albuminuria defined as urinary albumin excretion rate of 20 to 200 *μ*g/min.

TGF-*β*1 has a very wide range of activities *in vitro*. For example, TGF-*β* regulates important cellular functions such as rate of proliferation and production of extracellular matrix proteins by wide range of cell types. As a result of the wide range of activities attributed to the TGF-*β*, a number of groups have investigated whether circulating levels of TGF-*β*1 might be altered in various disease states. With only one exception, all of these studies agree that TGF-*β*1 is found in detectable levels in plasma from healthy human subjects [25]. TGF-*β* levels are unaltered for example in normal pregnancy [26].

Moreover, plasma TGF-*β*1 concentration markedly differed (by as much as 10-fold) in subjects suffering from various diseases, including autoimmune diseases, atherosclerosis and various cancers, compared with control subjects [25]. If such apathopysiological role of plasma TGF-*β*1 is proven, it could become both a prognostic indicator of future risk of disease and/or complications of disease and a target for therapeutic interventions.

**Figure 3:**
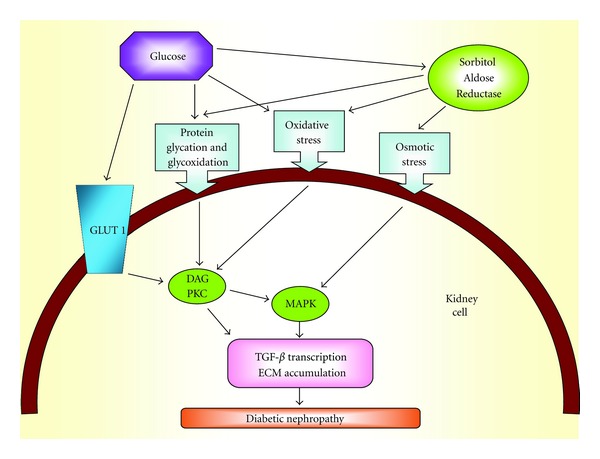
From hyperglycaemia to TGF-**β** transcription.

TGF-*β*1 plays a pivotal role in the extracellular matrix accumulation and in the pathogenesis of diabetic nephropathy (Figure 3). TGF-*β*1 may participate in the development and progression of diabetic micro- and macrovascular complications [27–29]. The association between TGF-*β*1 and cardiovascular disease in diabetic patients is controversial [30].

TGF-*β*1 is an important cytokine for the development of renal injury in patients with DM2 [31]. Higher serum levels of TGF-*β*1 were found in patients with DM2 [31–33]. In the study of patients with DM1 were also found alterations in level of circulating TGF-*β*1 [34, 35]. Elevated levels of circulating TGF-*β*1 were related to proliferative retinopathy and HbA1c [35]. AGEs play a critical role in diabetic nephropathy and vasculopathy and is associated with AGE deposition and receptor for AGE (RAGE) upregulation [18].

In our study the elevated levels of TGF-*β*1 in subjects with DM1 possibly indicate a tendency for renal and endothelial damage in such patients. Serum TGF-*β*1 can be probably one of diagnostic indicators for early diabetic vasculopathy. However, TGF-*β* correlated only with age and duration of DM1. There was not found any significant relation between circulating TGF-*β* and parameters of early and advanced glycation and oxidation. Nevertheless, diabetic nephropathy was absent in our diabetic patients.

## 5. Conclusions

The level of TGF-*β* in serum of young patients with DM1 (children and adolescents) was significantly higher in the comparison with healthy control. TGF-*β* correlates only with age and duration of DM1. There was not found any significant relation between circulating TGF-*β* and parameters of early and advanced glycation and oxidation. However, these results do not exclude the association between TGF-**β** and the onset of diabetic complications.

## Figures and Tables

**Table 1:** Clinical and biochemical parameters in all diabetic patients and healthy control.

Parameter	All patients withDM1	*n*	Controls	*N*
Age (r.)	15.2 ± 2.7	79	9.2 ± 4.9	31
Duration of DM (r.)	8.7 ± 3.0	79	—	—
UAER (*μ*g/min)	37.8 ± 116.8	74	—	—
FAM (mmol/L)	2.85 ± 0.50	75	1.62 ± 0.35^#^	29
HbA1c (%)	9.51 ± 1.90	79	5.0 ± 0.39^#^	21
s-AGEs (A.U.)*	67.85 (61.6; 76.4)	70	58.2 (52.0; 65.5)^#^	29
AOPP (*μ*mol/L)*	80.5 (44.9; 139.9)	59	58.5 (51.5; 66.9)^#^	12
LPO(nmol/mL)*	119 (100.3; 156.3)	48	99 (67; 106)^#^	11
TGF-*β* (ng/mL)*	7.1 (3.6; 12.6)	29	1.6 (0.8; 3.9)^#^	9

The results are presented as mean ± SD in normal distribution and as median (1st quartile, 3rd quartile) in data with abnormal distribution.*

^#^significant difference in comparison with DM1 patients.
